# Curcumin pharmacology and pharmacokinetics: current challenges and future perspectives in nanoemulsion delivery

**DOI:** 10.3762/bjnano.17.102

**Published:** 2026-10-29

**Authors:** Daniele De Campos, Marciane Zornita Bortolanza, Mohamed Fathi Abdallah, Jean-Marie Colet, Julia de Oliveira Primo, Carla Bittencourt, Najeh Maissar Khalil

**Affiliations:** 1 Chimie Interactions Plasma-Surface (ChIPS), Research Institute for Materials Science and Engineering, University of Mons, Av. Nicolas Copernic 3, 7000 Mons, Belgium; 2 Applied Nanostructured Systems Laboratory, Universidade Estadual do Centro-Oeste, UNICENTRO, Alameda Elio Antonio Dalla Vecchia, 838, 85040-167, Guarapuava, PR, Brazil; 3 Department of Food Science, Aarhus University, Agro Food Park 48, 8200 Aarhus, Denmark; 4 School of Medicine and Pharmacy, Biologie humaine et Toxicologie, University of Mons, Avenue du Champ de Mars, 7000 Mons, Belgium

**Keywords:** anti-inflammatory, antioxidant, bioavailability, curcumin, nanoemulsions, polypharmacology, polyphenols

## Abstract

Derived from the rhizome of *Curcuma longa L.*, curcumin has evolved from a traditional component of South Asian medicine into a prominent topic in modern pharmacology. Curcumin has a pleiotropic pharmacological profile with biological properties including antioxidant, anti-inflammatory, and anticancer effects. However, its clinical translation faces physiological and chemical challenges, characterized by low systemic bioavailability, instability at neutral to alkaline pH, and rapid metabolic clearance. This review examines the molecular mechanisms and biological barriers that limit its efficiency and explores nanotechnology as a strategy to optimize its pharmacokinetic profile. It focuses on nanostructured systems, emphasizing nanoemulsions, to improve stability and prolong systemic circulation. Finally, the current landscape of clinical studies and the prospects for using these nanosystems to manage inflammatory and oncological diseases are discussed to establish curcumin as a therapeutic alternative.

## Introduction

Curcumin, a natural polyphenolic molecule, is the primary biomolecule of the rhizome of *Curcuma longa L.* [1–2]. The use of this compound dates back over 5,000 years, originating in the civilizations of South Asia and the Indian subcontinent. The global expansion occurred through major trade routes. When Arab and European merchants encountered this “exotic” spice, it became known as “Indian saffron” [3–5]. In its place of origin, curcumin remains a symbol of purity and prosperity, with its yellow color historically associated with the sun and fertility [6]. In modern food technology, it functions as a versatile food additive (colorant E100), flavor enhancer, and natural preservative [7]. Its innate ability to combat free radicals assists in the oxidative stability of fats and oils, acting as a natural preservative in tropical climates before the advent of refrigeration [8–9]. Furthermore, the global recognition of its safety and efficiency has led to its implementation as a preferred additive, as it is considered healthier for the human body than synthetic alternatives [7].

Curcumin was initially isolated in 1815, and its molecular structure (C_21_H_20_O_6_) was determined in 1910 [10–11]. The “International Union of Pure and Applied Chemistry” (IUPAC) designates it as (1*E*,6*E*)-1,7-bis(4-hydroxy-3-methoxyphenyl)hepta-1,6-diene-3,5-dione. The molecular structure is characterized by two aromatic rings linked by a heptadienedione chain, which includes a β-diketone moiety, conferring the characteristics of a keto–enol tautomerism [12–13]. Due to these structural features, curcumin has attracted considerable scientific attention for its potential biological activities [14–15]. Notably, it demonstrates significant antioxidant and anti-inflammatory properties by neutralizing free radicals and inhibiting pro-inflammatory enzymes [16–17].

These properties have prompted investigations into its therapeutic applications across various diseases, with uses in dermatology and cosmeceuticals [4]. Research indicates its efficiency in enhancing skin nutrition and managing conditions such as eczema, where it acts as a cellular rejuvenation agent by mitigating tissue oxidative stress and offering protection against phototoxicity [18–19]. Despite this, the intense and characteristic yellow coloration of curcumin remains a limitation, especially for creams, gels, and depigmenting products that remain on the skin, where visible residue conflicts with expectations of sensory elegance and daily adherence. Recent formulation research aims not only to improve the solubility and cutaneous administration of curcumin but also to minimize surface staining through encapsulation and nanocarrier-based systems [20–21].

**Figure 1:**
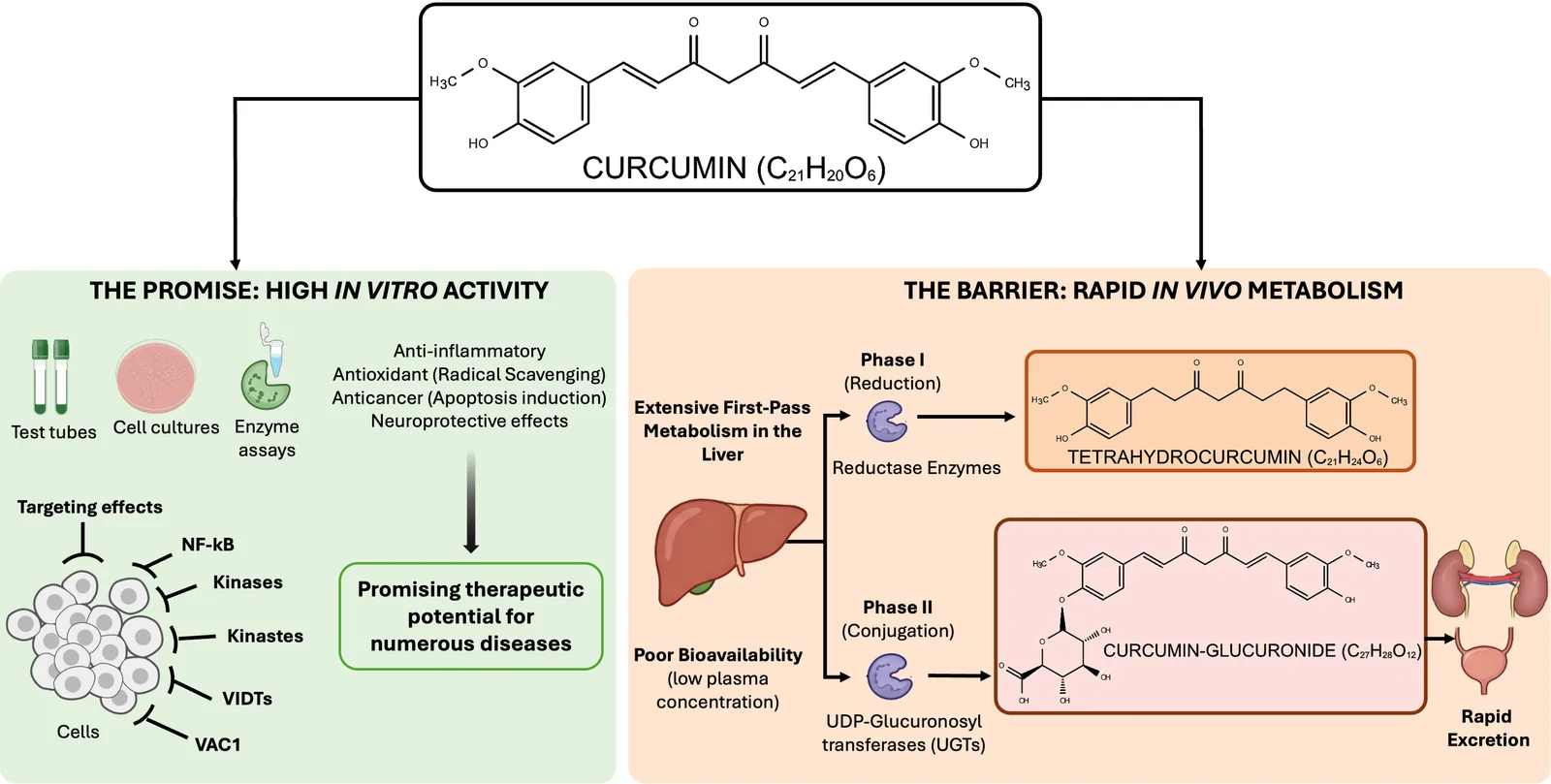
The pharmacological paradox of curcumin: high in vitro therapeutic potential versus rapid in vivo metabolism. The left panel (green) illustrates the multitarget activity of curcumin in simplified environments, highlighting its potent anti-inflammatory, antioxidant, and anticancer properties via the modulation of signaling pathways such as NF-κB and various kinases. The right panel (orange) depicts the pharmacokinetic barriers in complex organisms, characterized by extensive first-pass hepatic metabolism. Key metabolic pathways include Phase I reduction to tetrahydrocurcumin and Phase II conjugation to curcumin glucuronide, resulting in significantly increased water solubility, poor bioavailability, and rapid systemic excretion. Figure assembled by the authors using Microsoft PowerPoint. Graphical vector components were adapted from the NIAID BioArt Source (https://bioart.niaid.nih.gov/). All of the following content is not subject to CC BY 4.0. NIAID Visual & Medical Arts. (10/7/2024). Generic Cells. NIAID NIH BIOART Source. https://bioart.niaid.nih.gov/bioart/172. NIAID Visual & Medical Arts. (10/7/2024). BDBlood Vial. NIAID NIH BIOART Source. https://bioart.niaid.nih.gov/bioart/52. NIAID Visual & Medical Arts. (5/19/2025). Petri Dish with HEK293. NIAID NIH BIOART Source. https://bioart.niaid.nih.gov/bioart/653. NIAID Visual & Medical Arts. (10/7/2024). Eppendorf Tube. NIAID NIH BIOART Source. https://bioart.niaid.nih.gov/bioart/143. NIAID Visual & Medical Arts. (10/7/2024). Human Kidney. NIAID NIH BIOART Source. https://bioart.niaid.nih.gov/bioart/229. NIAID Visual & Medical Arts. (10/7/2024). Human Liver. NIAID NIH BIOART Source. https://bioart.niaid.nih.gov/bioart/230.

Although curcumin is widely recognized as a safe medicinal agent with significant therapeutic potential, its application in free form is hindered by several critical challenges. These include poor solubility in aqueous environments, rapid metabolic degradation, and low chemical stability [5]. These pharmacokinetic limitations drive the development of advanced pharmaco-technical strategies, including nanotechnology, to enhance bioavailability and maximize full therapeutic efficiency (Figure 1) [4].

While understanding the molecular mechanisms and pharmacokinetic limitations of free curcumin provides the essential biological rationale, bridging the translational gap requires equal focus on advanced formulation engineering. This review provides a comprehensive analysis of curcumin applications, emphasizing its pharmacological properties and the underlying mechanisms that contribute to its therapeutic potential in modern science. The literature search methodology is described in Supporting Information File 1. To establish the biological rationale for developing nanocarriers, subsections 1.1–1.4 describe the intrinsic molecular mechanisms and pleiotropic therapeutic properties of free (unencapsulated) curcumin, as demonstrated in standard cellular and molecular assays. Section 3 specifically evaluates how these active mechanisms integrate into nanoemulsions and related nanostructures.

## Review

### 1 Pharmacological properties and underlying mechanisms of curcumin

**Figure 2:**
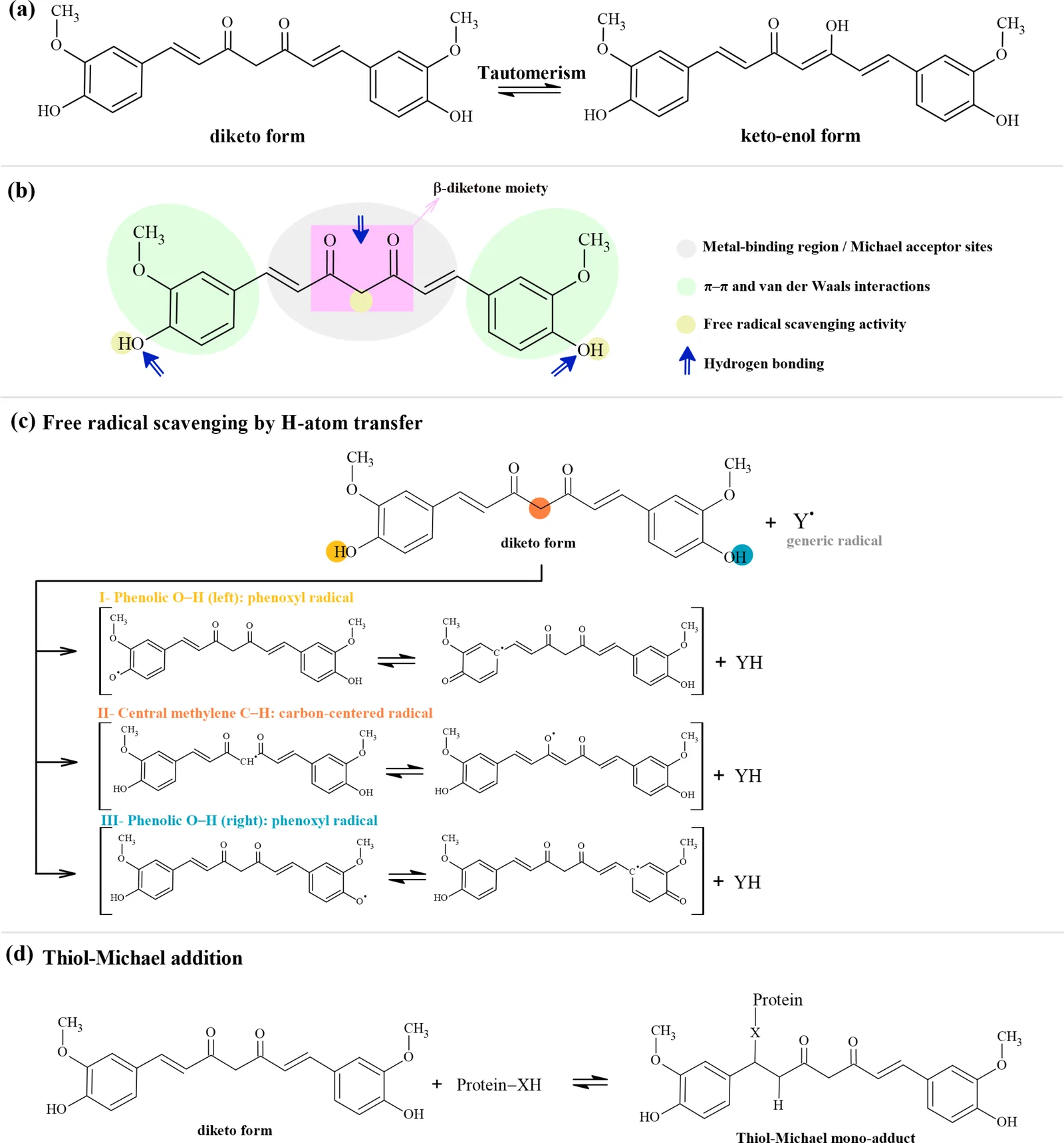
Structural features and representative chemical reactivity of curcumin. (a) Equilibrium between the neutral diketo form and one representative keto–enol tautomer. (b) Structural regions associated with metal binding, Michael acceptor activity, hydrogen bonding, aromatic interactions, and potential hydrogen-atom donation. (c) Alternative initial hydrogen-atom-transfer pathways involving a generic radical (Y•), which forms YH. Pathways I and III involve the symmetry-equivalent phenolic hydroxy groups and generate phenoxyl radicals; pathway II involves the central methylene group of the diketo form and initially generates a carbon-centered radical. Selected resonance contributors are connected by double-headed resonance arrows. Pathway contributions depend on the reacting radical and the medium; equal contributions are not implied. These chemical pathways underpin the compound’s ability to modulate diverse cellular signaling pathways. (d) Thiol-Michael addition.

The pharmacological efficiency of curcumin is closely associated with its unique chemical structure [22–23]. Its molecular architecture consists of two phenolic aromatic rings connected by a seven-carbon chain containing β-diketone groups, which confers significant chemical reactivity, primarily due to keto–enol tautomerism [12,24]. This structural arrangement allows curcumin to interact with a wide array of molecular targets and influence diverse cellular pathways, a characteristic commonly referred to as polypharmacology [25]. Curcumin undergoes keto–enol tautomerism between its neutral diketo and keto–enol forms, with their relative populations depending on the surrounding medium, including pH and solvent composition [26–28] (Figure 2a). Owing to molecular symmetry, two equivalent keto–enol tautomers are possible, differing in the position of the enolic proton and the associated double bonds [29]. The β-diketone portion is central to curcumin’s biological activity, acting as a Michael acceptor that enables covalent bond formation with cysteine residues in target proteins (Figure 2d), thereby allowing curcumin to act as a network modulator that reshapes pathological cellular microenvironments [30–31]. Despite its potential, the clinical application of curcumin has historically been complicated by low systemic bioavailability and extensive biotransformation [32]. Upon administration, the compound undergoes rapid hepatic and intestinal metabolism, involving the gradual reduction of the carbon chain and subsequent conjugation with glucuronic acid and sulfate [22,33]. Although parent curcumin levels in portal blood and systemic tissues often remain low, the observed in vivo effects are largely attributed to its active metabolites, such as tetrahydrocurcumin and hexahydrocurcumin. These secondary compounds retain significant antioxidant and anti-inflammatory properties, which effectively explain the therapeutic benefits observed even at low plasma concentrations of the parent molecule [34–35].

Curcumin simultaneously interacts with an extensive array of intracellular signaling cascades, most notably the nuclear factor kappa B (NF-κB) pathway [36]. As a principal regulator of inflammatory response and tumor progression, NF-κB inhibition is the basis of curcumin’s therapeutic activities. By preventing the translocation of NF-κB to the nucleus, the compound downregulates the expression of critical pro-inflammatory genes, including cyclooxygenase-2 (COX-2) and inducible nitric oxide synthase (iNOS) [4,37]. Furthermore, curcumin modulates other key pathways, such as PI3K/Akt, JAK/STAT, and MAPK, and frequently activates JNK/p38 signaling to induce programmed cell death in pathological cells without compromising healthy tissue [38–39]. These interconnected mechanisms create a synergistic effect in which reducing the inflammatory milieu effectively reinforces antioxidant capacity and mitigates the progression of chronic diseases, ranging from neurodegeneration to cancer [37,40]. Consequently, the role of curcumin is extended from a simple dietary supplement to a complex pharmacological agent with a broad and diverse spectrum of biological activities.

#### 1.1 Anti-inflammatory effects

This conformation of curcumin provides the chemical basis for interactions that inhibit critical enzymes in various biological processes (Figure 2b). Specifically, the arrangement of functional groups, such as hydroxy and methoxy groups on the aromatic rings, confers on curcumin the ability to inhibit the enzymatic activities of COX-2 and LOX [41–42]. This structural interaction directly influences the arachidonic acid cascade, a fundamental metabolic pathway in the biosynthesis of inflammatory lipid mediators. As a result of the simultaneous inhibition of COX-2 and LOX enzymes, significant reductions in prostaglandin and leukotriene production occur, respectively [42–43].

Beyond direct enzymatic interference, curcumin acts comprehensively at the transcriptional level, modulating the expression of genes encoding cytokines and other proinflammatory molecules essential for the immune response [17,40]. This modulation of the inflammatory response extends to the suppression of key chemotactic cytokines, such as monocyte chemoattractant protein and macrophage inflammatory protein. This control is exerted through the regulation of specific signaling pathways and the activity of secondary inflammatory mediators, demonstrating that the therapeutic potential of curcumin stems from its synergistic ability to suppress both the initiation and propagation of chemical mediators of inflammatory processes [17,44].

There is evidence that curcumin promotes the reduction of leukocyte, neutrophil, and eosinophil populations at the inflamed site [45–46]. This cellular effect correlates with protection against elevated serum levels of mediators such as phospholipase A2 and total proteins, reinforcing the significant role of curcumin in the management and prevention of chronic inflammatory pathologies through a multifocal action ranging from molecular stabilization to cellular immune response [47]. Furthermore, the suppression of these inflammatory mediators is intrinsically linked to the modulation of oxidative stress as many pro-inflammatory pathways are triggered by reactive species, thereby producing a synergistic antioxidant effect.

#### 1.2 Antioxidant properties

Curcumin is distinguished by its remarkable ability to neutralize excess reactive oxygen species (ROS) in in vivo and in vitro models [48]. This property is primarily attributed to its molecular structure, specifically to two fundamental structural regions, namely, the phenolic groups and the β-diketone moiety [12–13,49]. The two phenolic hydroxy (–OH) groups located at the extremities of the aromatic rings act as the main sites of antioxidant activity, donating hydrogen atoms to neutralize free radicals via a chain-breaking mechanism (Figure 2c) [12,50]. The resulting curcumin-derived radical exhibits high stability due to extensive resonance delocalization throughout the molecule’s conjugated system [51]. The adjacent methoxy (–OCH_3_) groups modulate and amplify the reactivity of the phenolic groups, thereby strengthening the overall antioxidant potential [52–53]. The central β-diketone segment complements this process by allowing curcumin to adopt a stable enol form, providing an additional labile hydrogen atom for radical scavenging [9,54]. In addition to direct neutralization, this moiety plays a critical role in the chelation of transition metal ions, such as iron (Fe^2+^/Fe^3+^) and copper (Cu^2+^) [54–55]. By binding to these ions, curcumin prevents them from catalyzing the formation of free radicals, offering a dual protection mechanism against oxidative stress [53,56].

Beyond its direct action, curcumin potentiates endogenous antioxidant defense systems [48,57]. It stimulates the activity of key antioxidant enzymes, including catalase, superoxide dismutase, and glutathione peroxidase [58–59]. Additionally, the compound modulates the Keap1–Nrf2 signaling pathway, a central regulator of the cellular antioxidant response [60]. Through the activation of Nrf2, curcumin contributes to the maintenance of redox homeostasis and supports the detoxification of intracellular ROS [61].

#### 1.3 Anticancer potential

Curcumin has demonstrated therapeutic potential against a wide range of oncological pathologies. Preclinical studies (cell and animal models) show curcumin’s action in many cancers including breast, prostate, lung, skin, gastrointestinal tract (including colon and pancreas), liver, brain, ovary, leukemias, and multiple myeloma [62–65]. Specific reviews highlight effects on lung cancer, on oral and gastrointestinal tumors with an emphasis on metastasis [66–67], and on hepatocellular carcinoma [68]. Human trials are much scarcer, but there are signs of benefit in premalignant oral lesions, advanced pancreatic cancer, and as an adjuvant in breast cancer under chemotherapy [69]. Curcumin modulates multiple pathways linked to tumor initiation, promotion, angiogenesis, and progression, such as NF-κB, STAT3, PI3K/Akt/mTOR, MAPK, Wnt/β-catenin, and RTKs [62,64,70–71]. It inhibits proliferation, induces apoptosis, blocks angiogenesis, reduces invasion/metastasis (MMPs, EMT), and regulates ncRNAs/miRNAs [64,66,69–71]. It also acts as an antioxidant in normal cells and as a pro-oxidant in tumor cells, increasing ROS and cell death [62].

Conventionally, curcumin exhibits very low oral absorption, rapid metabolism, and nearly undetectable serum levels, limiting its systemic efficiency [64,70,72]. Several studies focus on nanoformulations, derivatives, and co-administration with other phytochemicals (e.g., piperine or resveratrol) to increase solubility, bioavailability, and synergy with chemo/radiotherapy [64,70,73–77]. Notably, the compound exhibits dual functionality in radiotherapy, acting as both a radioprotective and a radiosensitizing agent. In healthy tissues, curcumin attenuates radiation-induced oxidative stress and DNA damage; concurrently, it increases the sensitivity of malignant cells to radiotherapy, thereby optimizing therapeutic efficiency [78–81]. Curcumin increases radiosensitivity in multiple tumor lines (glioblastoma, prostate, colon, cervix, lymphoma, C6 glioma) by elevating ROS and inhibiting DNA repair mechanisms [78,80,82], blocking NF-κB and anti-apoptotic genes (Bcl-2, Bcl-XL, IAPs), altering the Bax/Bcl-2 ratio in favor of apoptosis [83–84], and modulating p53, PI3K/Akt/mTOR, and MAPK (ERK, JNK, p38), promoting cell cycle arrest (G2/M) and programmed cell death [82,85]. The suppression of metastatic progression is achieved through curcumin’s interference at various stages of the metastatic cascade. The compound inhibits tumor angiogenesis by modulating the NF-κB signaling pathway. It regulates multiple molecular targets essential for cell growth and survival, including growth factors, kinases, and pro- and anti-apoptotic proteins [83–85].

Additionally, curcumin acts as a molecular checkpoint that restricts uncontrolled cell proliferation. This control is exerted through the downregulation of cyclin D1 expression, a key regulatory protein in cell cycle progression [86–87]. By suppressing cyclin D1 production, curcumin halts the transition from the growth phase to the DNA synthesis phase, effectively blocking cell division [88]. This antiproliferative property constitutes one of the most extensively studied mechanisms of its chemopreventive and adjuvant action, being fundamental for the suppression of persistent tumor growth [89–90].

#### 1.4 Neuroprotective effects

The neuroprotective properties of curcumin derive from the synergistic interaction between its antioxidant, anti-inflammatory, and anti-apoptotic mechanisms [91–92]. Due to its lipophilic nature, the molecule can cross the blood–brain barrier (BBB), enabling the exertion of direct neuroprotective effects on the cerebral parenchyma [93]. Curcumin mitigates oxidative stress and neuroinflammation by inhibiting critical mediators, such as NF-κB, COX-2, and iNOS, and by modulating intracellular apoptotic pathways [94]. Additionally, its molecular configuration allows for the modulation of enzymes such as CaMKII2 (a critical enzyme involved in neurotransmitter synthesis, synaptic organization, long-term plasticity, learning, and memory consolidation), amplifying its capacity for neuronal preservation [95–96].

In models of neurodegenerative diseases, such as Parkinson’s disease, curcumin has been shown to protect dopaminergic neurons and reduce systemic oxidative stress by regulating pro-apoptotic signaling pathways [91,97]. Similarly, in Alzheimer’s disease, the compound modulates cell death cascades and reduces the expression of pro-inflammatory cytokines, promoting neuronal survival. A relevant mechanism involves its influence on microglial polarization, favoring the transition from the pro-inflammatory M1 phenotype to the anti-inflammatory M2 phenotype [98–99]. This phenotypic shift not only reduces neuronal apoptosis but also facilitates tissue repair and preserves the structural integrity of the BBB. Curcumin further interferes with NLRP3 inflammasome activation and the consequent secretion of IL-1β, thereby modulating NF-κB signaling [100]. Such action protects the tissue against ischemic injuries, cerebrovascular accidents (strokes), and white matter damage [101]. Concurrently, curcumin elevates the activity of endogenous antioxidant enzymes, reinforcing cellular defense [102]. At the synaptic system level, curcumin modulates glutamate receptor activity, preventing excessive calcium influx and attenuating excitotoxicity-induced neuronal death [103–104].

In the context of diabetic neuropathy, curcumin exhibits therapeutic effects by actively promoting nerve regeneration [105–106]. The primary mechanism involves the increased expression of nerve growth factor (NGF), whose synthesis is typically suppressed under hyperglycemic conditions [107]. By elevating the availability of NGF, curcumin provides essential molecular stimuli for neuronal survival, growth, and reconnection [106,108]. Additionally, its antioxidant and anti-inflammatory properties protect peripheral nerve fibers from chronic metabolic stress, establishing a favorable cellular environment for the functional restoration of the nervous system [109].

While the diverse pharmacological actions described above highlight the therapeutic potential of curcumin, these benefits are rarely fully realized in clinical settings when the compound is administered in its native form. This discrepancy leads to the “pharmacological paradox” of curcumin, necessitating a deeper understanding of its pharmacokinetic limitations.

### 2 Challenges of bioavailability of free-form curcumin

Curcumin, despite its diverse therapeutic potential, faces significant challenges regarding its clinical application due to its remarkably low bioavailability when administered in its free form [110]. This limitation is primarily attributed to a combination of physicochemical barriers and efficient biological elimination mechanisms, which together result in only a minimal proportion of the administered dose reaching the intended target tissues in a bioactive state.

#### 2.1 Low water solubility and instability at physiological pH

A major challenge to the clinical application of curcumin lies in its intrinsic hydrophobic nature, which makes it nearly insoluble in water (solubility < 0.1 µg/mL at room temperature) [11,111]. Curcumin is a highly lipophilic polyphenol characterized by an extensive system of conjugated double bonds that favor crystalline stability and π–π stacking over aqueous interactions [96]. This lipophilicity is primarily driven by the two symmetric 4-hydroxy-3-methoxyphenyl rings, which provide a substantial non-polar surface area, the seven-carbon unsaturated heptadienedione linker, which serves as a hydrophobic bridge, and the methoxy groups (–OCH_3_) on the aromatic rings that decrease the overall polarity of the molecule relative to simpler phenolic compounds [24–25]. These features result in a high partition coefficient (log *P* ≈ 3.2), ensuring that the molecule remains sequestered in lipid-rich environments rather than dissolving in the aqueous gastrointestinal milieu [112].

The keto–enol tautomerism of the β-dicarbonyl moiety contributes to the hydrophobic character of curcumin through an intrinsic chemical mechanism [113]. In hydrophobic environments and most organic solvents, the enol form predominates. A strong hydrogen bond between the enolic hydroxy group and the adjacent carbonyl oxygen stabilizes this conformation. This intramolecular bonding effectively reduces the molecule’s ability to form stabilizing intermolecular hydrogen bonds with water molecules. As a result, the initial dissolution process within the gastrointestinal lumen, a critical step for epithelial absorption, is severely hindered [114]. This physical limitation is further compounded by curcumin’s extreme sensitivity to the pH. While the diketo tautomer remains relatively stable under the acidic conditions of the stomach (pH 1.5–3.5), the transition to the neutral or slightly alkaline environment of the small intestine and systemic circulation induces a structural shift [27,53]. At physiological pH, the enol moiety undergoes deprotonation, forming the enolate form. This conformation is chemically unstable and predisposes the heptadienedione chain to rapid hydrolytic cleavage and auto-oxidation [11,113]. These degradation processes lead to the formation of phenolic byproducts, such as feruloyl methane, vanillin, and ferulic acid, which exhibit significantly reduced polypharmacological activity compared to the parent compound [115–116]. Moreover, curcumin exhibits significant photochemical instability, with exposure to visible and ultraviolet light catalyzing its degradation into inactive products. This demands stringent light protection during both experimental handling and clinical administration [117]. Additionally, curcumin is highly vulnerable to oxidative damage induced by ROS, further contributing to its instability. These combined factors drastically reduce the availability of intact, bioactive curcumin for systemic distribution [116].

#### 2.2 Rapid hepatic and intestinal metabolism

**Figure 3:**
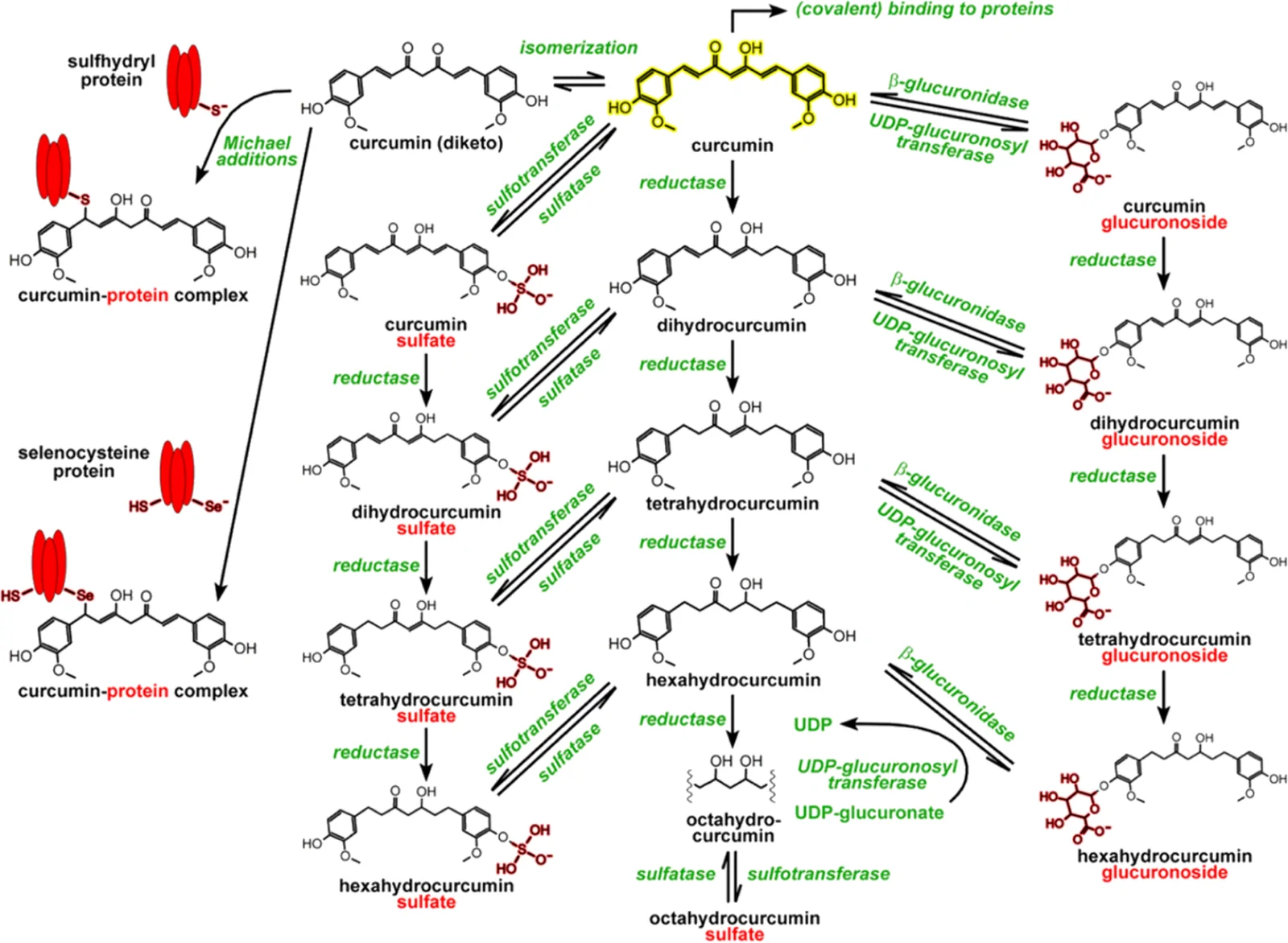
In 2014, Heger and colleagues provided an overview of curcumin metabolism. The figure shows the compound (shown in yellow), the chemical processes and enzymatic reactions (in green), and the additions to the curcumin molecule (in red). The figure was reprinted from [24], Pharmacological Reviews, vol. 66, by M. Heger; R. F. van Golen; M. Broekgaarden; M. C. Michel, “The Molecular Basis for the Pharmacokinetics and Pharmacodynamics of Curcumin and Its Metabolites in Relation to Cancer”, pages 222-307, Copyright (2013), The American Society for Pharmacology and Experimental Therapeutics, published by Elsevier, reprinted with permission from Elsevier. This content is not subject to CC BY 4.0.

Even when the initial solubility barrier is addressed, curcumin faces a significant metabolic challenge that is the primary obstacle to its systemic bioavailability and clinical efficiency [118]. This biotransformation process, first described by Wahlström and Blennow in 1978, involves a rapid, extensive metabolic cascade that occurs predominantly in the liver and intestinal mucosa, with the kidneys also playing a notable role in its elimination. The metabolic fate of curcumin is governed by two distinct enzymatic pathways: Phase I metabolism and Phase II conjugation. Phase I metabolism is characterized by the enzymatic reduction of the heptadiene double bonds within the heptadienedione chain of curcumin [32]. This reductive pathway involves the stepwise hydrogenation of the unsaturated C1–C2 and C6–C7 positions, mediated by nicotinamide adenine dinucleotide phosphate (NADPH)-dependent reductases located in the intestinal epithelium and hepatocytes [119–121]. The parent molecule is initially converted into dihydrocurcumin, which retains one double bond, and subsequently into tetrahydrocurcumin, in which both double bonds are fully saturated. Further enzymatic reduction of the ketone groups at the C3 and C5 positions produces secondary alcohols, yielding hexahydrocurcumin and, finally, the fully saturated octahydrocurcumin [24,122]. Following these Phase I reduction reactions, both the resulting metabolites and any residual parent curcumin undergo Phase II conjugation. This process involves the enzymatic attachment of polar groups, such as glucuronic acid or sulfate, to the phenolic hydroxy groups on the aromatic rings. These conjugation reactions are catalyzed by enzymes such as UDP-glucuronosyltransferases (UGTs) and sulfotransferases, resulting in the formation of highly water-soluble metabolites, including curcumin-glucuronide and curcumin-sulfate (Figure 3) [22,33]. While these conjugated forms enhance water solubility and facilitate excretion, they exhibit significantly reduced biological activity compared to the unmodified parent compound [122]. A critical factor in clinical efficiency is that most curcumin detected in human plasma exists in these conjugated forms. These metabolites are subject to rapid systemic elimination, primarily through biliary excretion via the gallbladder, as well as through fecal and urinary excretion. This rapid elimination further limits the bioactive concentration of curcumin available for therapeutic action in systemic circulation [118].

This rapid metabolic turnover explains the “curcumin paradox”. It has been reported that approximately 60% of an oral dose of curcumin is absorbed across the intestinal epithelium, but, due to extensive first-pass metabolism and interactions with gastrointestinal mucus, detectable levels of biologically active aglycone in portal blood and peripheral tissues remain negligible, often below 20 µg per tissue [35,123]. Ultimately, the challenge lies in ensuring that the biologically active form of curcumin reaches target tissues in sufficient concentrations to exert its therapeutic effects, a requirement that free-form curcumin consistently fails to meet due to its rapid metabolic conversion and elimination.

### 3 Advanced strategies for improving the therapeutic efficiency of curcumin

#### 3.1 Nanotechnology-based delivery systems

To address the challenges posed by the low bioavailability of curcumin, nanotechnology has emerged as a promising solution. The development of curcumin-based nanoparticles seeks to improve physicochemical stability, enhance solubility, increase cellular uptake, and enable controlled release [124–125]. These advanced delivery systems facilitate targeted delivery to specific cells, such as tumor cells, while simultaneously minimizing systemic toxicity and optimizing the pharmacokinetic and therapeutic properties of curcumin [126].

Pharmaceutical nanotechnology is rooted in the manipulation of materials at the nanoscale, a concept first envisioned by Richard Feynman in 1959 [127]. It is defined as the engineering and utilization of matter at the nanometric scale, allowing for the exploration of unique physicochemical phenomena that arise at this level to develop novel drug delivery systems [128–129]. A key advantage of this technology lies in the substantial increase in surface area-to-volume ratio achieved by reducing particle size to the nanoscale. This characteristic amplifies surface reactivity and enhances the solubility and dissolution rate of hydrophobic compounds like curcumin within aqueous environments. The augmented surface area permits greater interaction with a solvent, while the reduction in particle size may alter surface energy. Depending on the material properties of the particles, this may render dissolution more thermodynamically favorable. For hydrophobic substances such as curcumin, supplementary modifications, such as hydrophilic coatings, functional groups, or surfactants, are frequently necessary to improve dispersion and enhance apparent solubility in aqueous media [130]. Furthermore, by associating a drug molecule with a nanoparticle, targeted delivery, reduced renal filtration due to increased molecular size, and lower concentrations of the therapeutic agent can be achieved. Collectively, these factors significantly improve the therapeutic index while reducing systemic toxicity [131].

Improved solubility is a critical determinant of oral absorption because poorly water-soluble compounds must first dissolve in gastrointestinal fluids before they can permeate the intestinal epithelium [132–133]. Accordingly, formulation strategies such as particle size reduction, incorporation of surfactants or hydrophilic carriers, and the application of nanotechnology enhance surface area, wettability, and apparent solubility, thereby accelerating dissolution and increasing intestinal uptake [134–135]. In addition to improving dissolution behavior, nanostructured delivery systems can further promote absorption by protecting the active compound from premature degradation, facilitating transport across mucosal and epithelial barriers, reducing the impact of efflux transporters and favoring lymphatic uptake, which can partially circumvent first-pass metabolism [136–137]. The resulting increase in absorption generally leads to greater systemic exposure, typically reflected by higher plasma area under the concentration–time curve (AUC), increased maximum plasma concentration (*C*_max_), and, in some cases, prolonged residence time [138]. Curcumin illustrates this relationship particularly well since its intrinsically low aqueous solubility and poor oral bioavailability limit the attainment of effective therapeutic concentrations. Nanoformulation approaches have been shown to improve both the pharmacokinetic profile and biological performance of curcumin by enhancing solubility and oral bioavailability [139–140]. These pharmacokinetic gains are frequently accompanied by improved therapeutic outcomes, including stronger anticancer, anti-inflammatory, and other pharmacodynamic effects, while in some cases also allowing for lower effective doses and reduced systemic toxicity [75,135,140–141].

In pharmaceutical nanotechnology, nanocarriers function as efficient drug delivery systems, capable of encapsulating high concentrations of active compounds, protecting them from premature enzymatic degradation, and enabling controlled release at specific sites of action [142–143]. These nanocarriers play a critical role in shielding molecules from enzymatic degradation, modulating pharmacokinetics by extending systemic circulation time, and promoting site-specific targeting, thereby minimizing toxicity in healthy tissues [144]. The range of nanomaterials applicable in healthcare is vast, including nanoparticles, micelles, dendrimers, and lipid-based systems such as nanoemulsions [126]. Nanoemulsions have demonstrated exceptional efficiency in delivering lipophilic natural compounds like curcumin. Despite their significant therapeutic potential, the transition of nanotechnology-based drug delivery systems from laboratory research to clinical application faces substantial challenges. These include ensuring industrial scalability, achieving batch-to-batch reliability, maintaining long-term physicochemical stability, and establishing the biological safety of the final product. Furthermore, the economic feasibility of production processes remains a critical factor influencing the commercial viability of nanomedicines [124–125]. Consequently, rigorous research on the design, characterization, and surface modification of these nanostructures is essential for developing effective therapeutic applications, particularly for the treatment of chronic diseases.

In summary, the performance of nanocarrier-mediated delivery results from sequential steps that lead to pharmacological activity. Encapsulation in nanocarriers overcomes, first, the intrinsic limitation of solubility by disposing of the lipophilic drug in the oily core and increasing the effective surface area. Second, the nanometric size of the droplets and the fluidization of the membrane mediated by surfactants facilitate active epithelial absorption via endocytic pathways. Third, the protection of the active molecule prevents premature hydrolytic cleavage at physiological pH and avoids rapid Phase I reduction and Phase II glucuronidation, drastically increasing systemic exposure (higher *C*_max_ and plasma AUC of the intact aglycone). Finally, this sustained systemic circulation allows for active accumulation in the target tissue (via enhanced permeability and retention (EPR) effect or ligand targeting), converting enhanced bioavailability into preclinical and clinical models (Table 1).

**Table 1:** Studies explaining the main pharmacokinetic findings related to curcumin carried by nanoemulsions.

Nanoemulsion type	Biological model	Pharmacokinetic discoveries	Reference

nanoemulsions by spontaneous emulsification technique	in vitro (neuroblastoma SH-SY5Y) and in vivo (Sprague Dawley rat)	provided an extended half-life, prolonged systemic circulation, high cellular uptake, and superior neuroprotection against copper overload	[145]
nanoemulsions by high-shear homogenization followed by a microfluidization process	in vivo (Sprague Dawley rats)	enhanced total bioavailability, along with increased accumulation in target organs (liver) and brown adipose tissue.	[146]
nanoemulsions by ultrasonication	in vivo (female CD-1 mice)	increased total bioavailability (AUC) and *C*_max_	[147]
nanoemulsions by vortex homogenization	in vivo (CD2F1 mice)	increased total bioavailability (AUC), *C*_max_, and circulating bioavailability	[173]

#### 3.2 Nanoemulsions

Among various colloidal delivery systems, nanoemulsions stand out as a versatile platform for the systemic delivery of lipophilic polyphenols like curcumin (Table 2). Nanoemulsions are heterogeneous colloidal systems composed of two immiscible liquids, typically an oil phase and an aqueous phase, stabilized by a layer of surfactants and co-surfactants [148]. The droplets in these systems typically range from 20 to 200 nm in size, though some studies extend this range to 500 nm [149–150]. Based on the arrangement of the phases, nanoemulsions can be classified as oil-in-water (O/W), water-in-oil (W/O), or multiple emulsions, in which oil and water droplets form multiple layers [150]. Although nanoemulsions are thermodynamically unstable, they are kinetically stable, meaning they resist phase separation for extended periods. This stability is achieved through the small droplet size and the energy barrier created by surfactants, which delays destabilization processes such as coalescence, flocculation, and Ostwald ripening [149]. Incorporating curcumin into these nanodroplets provides a protective environment that shields the hydrophobic compound from degradation in aqueous environments, significantly enhancing its solubility and permeability [131,151].

**Table 2:** Summary of curcumin-loaded nanoemulsions and biological evaluations.

Nanoemulsion type	Year	Biological tests	Reference

curcumin-loaded water–oil–water double emulsion	2026	cellular toxicity (Hep G2 and fibroblast cells (L929))	[167]
curcumin-loaded O/W by high-energy ultrasonication	2025	antioxidant activity (DPPH assay), cytotoxicity (A549 cells), and efficiency were assessed in BALB/c mice through histological analysis	[130]
curcumin-loaded O/W homogenized by sonication	2025	antioxidant activity assays (DPPH and FRAP methods), cytotoxicity (fibroblasts) and skin penetration studies	[21]
curcumin-loaded O/W by spontaneous emulsification	2025	cell viability analysis and cellular uptake analysis (SH-SY5Y cell line)	[145]
curcumin-loaded O/W by high-energy ultrasonic emulsification	2025	antioxidant activity (DPPH)	[168]
curcumin-loaded O/W emulsion by ultrasonic processing	2024	antibacterial activity (*Acinetobacter baumannii* ATCC 19606, *Escherichia coli* ATCC 13846, *E. coli* ATCC 25922, *Enterococcus faecalis* ATCC 29212, *Klebsiella pneumoniae* ATCC 13883, *K. pneumoniae* ATCC 700603, *Pseudomonas aeruginosa* ATCC 27853, *Staphylococcus aureus* ATCC 25923, and *S. aureus* ATCC 33591) and antibiofilm activity (*Klebsiella pneumoniae*)	[169]
curcumin-loaded oil–water–oil by ultrasonication	2024	cytotoxicity (MTT) and anti-melanogenesis activity (B16F10 cells)	[170]
curcumin-loaded O/W by magnetic stirring	2023	anti-fungal activity (*Candida Albicans*) and in vitro permeation study (dialysis membrane)	[171]
curcumin-loaded O/W by ultrasonication	2023	anti-inflammatory (Sprague Dawley rats)	[172]

Beyond solubilization, nanoemulsions improve the therapeutic efficacy of curcumin by addressing its poor bioavailability and complex pharmacokinetics. These systems prevent premature degradation of curcumin, enable sustained release, and ensure that a higher concentration of the bioactive compound reaches target tissues [152–154]. Additionally, surface modifications, such as polyethylene glycol (PEG) coatings, are often employed to extend the systemic circulation time of curcumin nanoparticles by reducing elimination by the reticuloendothelial system. This prolongs the presence in the bloodstream, allowing the drug to leverage the EPR effect for targeted accumulation in tumor tissues [155].

Producing stable nanoemulsions requires precise control over factors such as particle size, surface charge, and surfactant selection [156–157]. High-energy methods, such as high-pressure homogenization, microfluidization, and ultrasonication, rely on intense mechanical forces to overcome the interfacial tension and break down large droplets. High-pressure homogenization produces nanometric particles by passing a coarse emulsion through a restricted orifice under extreme pressure (35 to 350 bar), where the simultaneous application of intense turbulence, hydraulic shear, and cavitation results in the formation of minute particles [158–159]. Microfluidization uses microchannels and the frontal collision of two high-velocity macroemulsion streams to produce narrower particle-size distributions and smaller droplet diameters with minimal surfactant concentration [160–161]. In contrast, ultrasonication operates through acoustic cavitation, the formation, growth, and implosive collapse of microscopic bubbles, which creates localized high-pressure “hot spots” that disrupt the oil–water interface [162].

Alternatively, low-energy methods exploit the internal chemical energy of the components, inducing phase transitions by varying parameters such as temperature or composition [163]. The phase inversion temperature method uses a thermal gradient to alter the spontaneous curvature of non-ionic surfactants; for instance, increasing temperature dehydrates polyoxymethylene groups, making them more lipophilic and inverting an O/W emulsion into a W/O emulsion. Rapid temperature shifts upon reaching the hydrophilic–lipophilic balance equilibrium achieve the necessary kinetic stabilization [164]. Similarly, the phase inversion composition method relies on the progressive addition of water to alter surfactant hydration and interfacial curvature, eventually forming nanometric droplets [165]. While low-energy methods are more energy-efficient and suitable for thermally sensitive compounds, they often require higher surfactant concentrations, which can limit their use in certain pharmaceutical or food-grade formulations [166].

In in vivo models, various nanoemulsion formulations have consistently improved the pharmacokinetic profile of the molecule, with increases in parameters such as AUC, *C*_max_, and relative bioavailability [173]. Complementarily, the development of organogel-based nanoemulsion formulations has also shown promising results, yielding a significant gain in total bioavailability [147]. To modulate the absorption and distribution of curcumin, the formulation of emulsions rich in medium-chain triglycerides has proven to be a safe alternative. In vivo assays showed that this lipid matrix enables accumulation of free, active forms in target organs, notably not only in the liver but also in adipose tissue. This efficient transport across biological barriers highlights how the nanocarrier can target the molecule to exact sites of action, significantly enhancing the therapeutic efficacy of the formulation [146]. In this context, the physicochemical characteristics of nanoemulsions, particularly their nanometric droplet size and lipophilic nature, play a decisive role not only in sampling, but also in the efficacy of tissue distribution of the active compound. This type of carrier system considerably facilitates permeation through the intestinal epithelium, protecting the molecule and ensuring its structural integrity throughout the diffusion process [145].

### 4 Challenges and future directions in clinical research

Although curcumin nanoemulsions demonstrate enhanced solubility and show significant results in preclinical studies, there remains a need to standardize key aspects essential for their optimization and human application, such as dosage, formulation composition, route, and duration of administration. Accordingly, while curcumin nanoemulsions can be obtained through simple methods and low-cost reagents, scalability challenges and regulatory hurdles in various countries must be addressed to enable their broad clinical and industrial application [174–175].

Despite the substantial advancements in curcumin-loaded nanocarriers, a significant translational gap remains between preclinical success and widespread clinical implementation. Currently, most clinical trials continue to rely on traditional pharmaceutical forms, such as tablets and capsules, reflecting the persistent difficulties in converting the theoretical advantages of nanotechnology into practical bedside applications [111]. A primary obstacle in this transition is the inconsistent observation of the EPR effect in human patients [176]. While animal models often show robust passive accumulation of nanoparticles in tumors, human malignancies exhibit high inter- and intrapatient heterogeneity, in which dense stromal barriers and variable vascular pressures frequently prevent nanomedicines from reaching the target site in therapeutic concentrations [177–178]. Consequently, the reliance on passive targeting alone has proven insufficient for many complex pathologies, necessitating a shift toward more sophisticated, ligand-mediated active targeting strategies.

Nanoparticle stability remains a critical issue, particularly due to droplet coalescence and phase separation, as well as the inherent instability of curcumin, which can limit the development of viable formulations for long-term storage [179]. Furthermore, transitioning from laboratory-scale production to industrial-scale manufacturing, one of the primary challenges in obtaining commercially viable curcumin-loaded nanoemulsions, requires robust equipment, protocols, and processes capable of yielding large production volumes while maintaining essential physicochemical parameters of nanoemulsions, such as particle size, encapsulation efficiency, and zeta potential, across different production batches. The clinical adoption of curcumin-targeted materials is hindered by a lack of standardized production protocols and rigorous quality control measures. Scaling up the synthesis of nanoemulsions from laboratory to industrial levels often results in batches with inconsistent particle sizes and surface charges, which directly impacts pharmacological behavior and stability [20,180].

To address and overcome these challenges, approaches based on the standardization of manufacturing processes and the maintenance of stability in accordance with regulatory guidelines [181] are required. Furthermore, toxicological studies, particularly those conducted over extended periods, are necessary to confirm that long-term safety concerns also remain at the forefront of clinical research, particularly regarding the potential for long-term tissue accumulation and the unknown systemic toxicity of insoluble excipients used in nanocarrier fabrication [182]. There is an urgent need for more comprehensive research in nanotoxicology to evaluate how these materials interact with the human body over extended periods, including their potentially adverse effects on the gut microbiota and the stability of the nanoformulations under diverse physiological conditions. Therefore, formulation standardization, development methods and processes, and safety in clinical evidence are yet to be obtained and are fundamental aspects to enable the transition of curcumin nanoemulsions from basic research to clinically validated products.

## Conclusion

This review has mapped the complex trajectory of curcumin, a pleiotropic polyphenol that stands at the intersection of traditional medicine and advanced drug delivery. We began by elucidating how its chemical structure, specifically the 3-methoxy phenolic groups and the β-dicarbonyl moiety, grants it a unique polypharmacology, allowing curcumin to modulate diverse signaling pathways such as NF-kB and Nrf2. We then described the “curcumin paradox”, that is, the reality that the low solubility, pH instability, and aggressive first-pass metabolism into inactive conjugates naturally throttle its immense therapeutic potential.

The second half of our analysis shifted to nanotechnology-based approaches. By employing nanoemulsions and self-assembling systems, we can shield the molecule from enzymatic degradation. These systems do more than just improve solubility; they employ mechanisms such as the EPR effect and active ligand-targeting to ensure that the bioactive aglycone reaches pathological sites while minimizing systemic toxicity.

Ultimately, the narrative of curcumin is shifting from a struggle with bioavailability to a triumph of engineering. As we navigate the clinical landscape of 2026, the therapeutic efficacy and clinical utility of this compound are independent of its botanical origin, but depend on the precision of the vehicle that carries it. The transition requires us to overcome the final hurdles of human stromal barriers and industrial standardization. Advancing the clinical substantiation of curcumin through rigorous trials is imperative to justify its transition from a molecule with significant biological properties to a validated therapeutic drug, particularly in the management of neoplasms and chronic inflammatory pathologies. By enhancing physicochemical stability and prolonging systemic half-life, nanostructured systems provide adequate concentrations for potential pharmacological activity. Therefore, conducting and validating advanced clinical trials is essential to confirm the safety and efficacy of these formulations.

A particularly innovative frontier is the emerging recognition of curcumin as a structural “builder” for its own delivery systems. By exploiting its ability to self-assemble via noncovalent interactions, such as stacking and hydrophobic interactions, researchers have developed carrier-free nanoparticles with drug-loading capacity. This shift toward carrier-free technology minimizes the use of potentially toxic inert excipients, representing a significant advancement in high-density formulation design.

## Supporting Information

File 1 Literature search methodology.

## Data Availability

Data sharing is not applicable as no new data was generated or analyzed in this review.
