# Supplementary material for: Curcumin pharmacology and pharmacokinetics: current challenges and future perspectives in nanoemulsion delivery
Source: Beilstein J Nanotechnol. 2026 Oct 29;17:1488–504. doi: 10.3762/bjnano.17.102 (PMC13725486; doi:10.3762/bjnano.17.102)
Supplement: Supplementary file 1 [file Beilstein_J_Nanotechnol-17-1488-s001.pdf]

BEILSTEIN JOURNAL OF NANOTECHNOLOGY

Supporting Information

for

Curcumin pharmacology and pharmacokinetics: current challenges and future perspectives in nanoemulsion delivery

Daniele De Campos, Marciane Zornita Bortolanza, Mohamed Fathi Abdallah, Jean-Marie Colet, Julia de Oliveira Primo, Carla Bittencourt and Najeh Maissar Khalil

Beilstein J. Nanotechnol. **2026,** *17,* 1488–1504. doi:10.3762/bjnano.17.102

Literature search methodology

License and Terms: This is a supporting information file under the terms of the Creative Commons Attribution License (https://creativecommons.org/licenses/by/4.0). Please note that the reuse, redistribution and reproduction in particular requires that the author(s) and source are credited and that individual graphics may be subject to special legal provisions.
The license is subject to the *Beilstein Journal of Nanotechnology* terms and conditions: (https://www.beilstein-journals.org/bjnano/terms)

Search Methodology

This study is structured as a narrative review focusing on the chemical, biological, and pharmacokinetic properties of curcumin, as well as on recent nanotechnological strategies developed to overcome barriers to its systemic bioavailability. Special emphasis was placed on nanoemulsion-based systems to enhance aqueous solubility, chemical stability, and therapeutic efficacy. The objective was to compile and evaluate studies detailing the polypharmacological mechanisms of curcumin, preparation methodologies, and prospects for clinical translation.

A literature search was conducted using PubMed, Scopus, Web of Science and ScienceDirect databases. The search strategy was based on combinations of relevant keywords, including curcumin, Curcuma longa, keto-enol tautomerism, bioavailability, pharmacokinetics, first-pass metabolism, anti-inflammatory, antioxidant, anticancer, neuroprotective, nanotechnology, nanoemulsions, methods for producing nanoemulsions, and clinical translation. The study prioritized peer-reviewed articles published through 2026. Since this is a review, there are well-established concepts regarding curcumin; therefore, we kept the review up to date while also drawing on foundational literature on curcumin isolation, chemical characterization, and primary metabolic pathways. Original research articles, in vitro and in vivo preclinical studies, and relevant review articles were selected for this synthesis.

Search Strategy and Reproducibility

To improve transparency and methodological clarity, representative Boolean search strings were constructed and applied across the selected databases. The following combinations were used: (“Curcumin” OR “diferuloylmethane” OR “Curcuma longa”) AND (“pharmacokinetics” OR “bioavailability” OR “metabolism” OR “first-pass metabolism” OR “tautomerism”) AND (“pharmacology” OR “anti-inflammatory” OR “antioxidant” OR “anticancer” OR “neuroprotective”) AND (“nanotechnology” OR “nanoemulsions” OR “nanoparticles” OR “nanocarriers” OR “drug delivery systems”) AND (“high-energy methods” OR “low-energy methods” OR “clinical trials”). These search strings were adapted to the syntax requirements of each database to optimize retrieval while maintaining specificity. The final literature search was conducted in January 2026.

Criteria for Inclusion and Exclusion

Inclusion criteria were (i) studies focused on the structural, chemical, and biological mechanisms of curcumin across anti-inflammatory, antioxidant, anticancer, or neuroprotective

models, (ii) research evaluating pharmacokinetic challenges, metabolic degradation, or nanotechnological approaches (specifically nanoemulsions) to enhance systemic stability and bioaccessibility, and (iii) peer-reviewed original research articles, meta-analyses, and high-impact review papers published in English. The exclusion criteria were: (i) studies investigating synthetic analogs of curcumin without a direct comparison to the original curcumin; (ii) articles that did not provide a clear understanding of the mechanisms or experimental evidence relevant to curcumin; and (iii) preprints not subject to peer review, conference abstracts without a complete methodology, editorials, and commentary articles.
